# Respond to the editor regarding “The investigation of effect on foot plantar massage on functional recovery in older adults with general surgery, randomized clinical trial”

**DOI:** 10.1007/s40520-024-02801-y

**Published:** 2024-08-09

**Authors:** Asuman Saltan, Selda Mert, Önder Topbaş, Beyza Aksu

**Affiliations:** 1 Department of Physiotherapy and Rehabilitation, Faculty of Health Sciences, Yalova University, Yalova, Turkey; 2 Department of Nursing, Faculty of Health Sciences, Kırşehir Ahi Evran University, Kırşehir, Turkey; 3 Department of Medical Services and Techniques, Kocaeli Vocational School of Health Services, Kocaeli University, Kocaeli, Turkey; 4 Kocaeli Vocational School of Health Services, Department of Health Care Services, Kocaeli University, Kocaeli, Turkey

Dear Editor, We thank Ma H and Wang X for their interest in our article. The aim of this article is to investigate the effect of classical foot massage on kinesiophobia and functional level in older adults after general surgery. This study is the first of its kind in its field. As there are no previous studies, the authors’ comments are very important for future studies.

The aim of this study is to create a rehabilitation protocol not only for orthopaedic or cardiovascular surgery, but also for fear of falling and changes in functional level after abdominal/ general surgery in older adults. From this point of view, regarding the first comment, the inclusion criteria were tried to be very general. This is because the cause of kinesiophobia in older adults has not been clearly identified [1]. Many factors are involved, ranging from economic status and gender to pain [1, 2]. For this reason, the inclusion and exclusion criteria were chosen to be as close as possible to the literature (as mentioned in the article). The literature indicates that there is a need for studies on this topic [1]. We believe that our study contributes to the literature with its inclusion and exclusion criteria [1–3].

Secondly, the massage used in this study was performed as described in the Methods Sect. (3). In the literature, the application of massage to older adults is usually aimed at increasing soft tissue mobility and affecting local blood circulation to make the person feel better [4]. In this study, no special method (reflexology or acupuncture point stimulation, etc.) was applied under the foot. According to this study, in future studies the massage techniques can be more individualised and the technique can be transferred to kinesiophobia and functional level analysis in older adults.

Thirdly, the situation regarding measurement times in this study is explained in the limitations section. Discharge times were similar between groups, but not within groups. Therefore, we felt that we could not evaluate the short-term effect of massage depending on the measurement time. According to the literature, the mobilisation or discharge process of individuals may differ depending on the surgical procedure [5]. Therefore, the investigation of the short or long effect of massage in this article is left to future studies. In this study, we wanted to investigate whether or not this type of application has an effect. It was found that foot massage applied to older adults after general surgery had a positive effect [3].

This study is a first step towards choosing a method to implement in the rehabilitation process to kinesiophobia and functionality for older adults after general surgery. We agree with Ma H and Wang X that there is a need to increase the number and quality of future studies in this area.

## Data Availability

No datasets were generated or analysed during the current study.
